# From policy to practice: a qualitive study exploring the role of community health workers during the COVID-19 response in Sierra Leone

**DOI:** 10.1186/s12913-023-10272-6

**Published:** 2023-11-09

**Authors:** Lansana Hassim Kallon, Joanna Raven, Haja Ramatulai Wurie, Wesam Mansour

**Affiliations:** 1 College of Medicine and Allied Health Sciences, University of Sierra Leone, Freetown, Sierra Leone; 2 Department of International Public Health, Liverpool School of Tropical Medicine, Pembroke Place, Liverpool, L3 5QA UK

**Keywords:** Sierra Leone, Community health workers, CHWs, COVID-19

## Abstract

**Background:**

During the COVID-19 pandemic, community health workers (CHWs) were required to help their communities respond to the outbreak in Sierra Leone. The Government of Sierra Leone released a policy that provided an interim guidance on the specific role of CHWs during the pandemic including support required to maintain continuity of routine and essential services during the COVID-19 response. This study explores how CHWs adapted their roles during the COVID-19 pandemic in Sierra Leone and the support they received from families, communities, and the health system.

**Methods:**

A qualitative exploratory study was conducted in two districts in Sierra Leone. We conducted eight key informant interviews with district and community level managers and leaders and four focus group discussions with CHWs. Thematic data analysis and synthesis were guided by the interim guidance released by the Government of Sierra Leone at the onset of the COVID-19 pandemic and supported by NVivo 11.

**Results:**

CHWs quickly took on COVID-19 frontline roles which included surveillance, contact tracing, social mobilization, and provision of psychosocial support. CHWs were trusted with these responsibilities as they were recognized as being knowledgeable about the community, were able to communicate effectively with community members and had experience of dealing with other outbreaks. Despite the release of the interim guidance aimed to optimize CHW contribution, motivate CHWs, ensure continuity of core and essential community-based services alongside COVID-19 services, CHWs faced many challenges in their work during the pandemic including heavy workload, low financial remuneration, lack of mental health support, and shortages of protective equipment, communication and transportation allowances. However, they were generally satisfied with the quality of the training and supervision they received. Support from families and communities was mixed, with some CHWs experiencing stigma and discrimination.

**Conclusion:**

During the COVID-19 pandemic, CHWs played a critical role in Sierra Leone. Although, a policy was released by the government to guide their role during the crisis, it was not fully implemented. This resulted in CHWs being overworked and under supported. It is important that CHWs are provided with the necessary training, tools and support to take on their vital roles in managing health crises at the community level. Strengthening the capacity of CHWs will not only enhance pandemic response, but also lay the foundation for improved primary health care delivery and community resilience in the face of future health emergencies.

**Supplementary Information:**

The online version contains supplementary material available at 10.1186/s12913-023-10272-6.

## Background

The health workforce is critical to achieving Universal Health Coverage [1]. However, there is a global shortage of formal health workers, and many countries in the global South, particularly fragile and shock-prone settings, increasingly look to a range of Community Health Workers (CHWs) to fill this gap [2, 3]. In epidemic emergencies such as Ebola and cholera outbreaks, CHWs and other community volunteers in many countries in the sub-Saharan African region bridge the gap between local communities and the formal health system [4]. They have provided critical frontline services such as contact tracing, community sensitization, as well as maintained routine health services within their scope of work [5–7].

The COVID-19 pandemic has further demonstrated the value of CHWs in supporting the response at the community level [8]. It has highlighted the strategic position CHWs manage between communities and health systems, often in contexts where mistrust exists between service users and the formal health system [8, 9]. Recent studies highlighted clear roles and responsibilities, training, supportive supervision, work satisfaction, health and well-being as critical in maintaining CHWs’ roles during the response to emergencies such as the COVID-19 pandemic [5, 10].

Sierra Leone has shortages of key and formal healthcare workers. A revised national Community Health Worker Policy was launched in February 2017^11^ and rolled out nationwide with 15,000 CHWs, selected by their communities and trained to provide a basic package of services at the community level which include: reproductive, maternal, neonatal and child health services; preventive and therapeutic services for common child illnesses such as malaria, diarrhoea and pneumonia, and nutritional services; and events reporting for childbirth, maternal deaths, and disease outbreaks [11].

There have been studies investigating the roles and experiences of CHWs in Sierra Leone [12–14]. They found that CHWs are often trusted and respected members of their communities who are bridges between their communities and the health system and are able to reach the most marginalised in their communities. However, they do not always have the support that they need from the health system, community, and family to fulfil their critical role [12, 13]. In addition, CHW experiences are influenced by context specific gender norms and relations which can challenge and support their work [6, 14].

During the COVID-19 outbreak in Sierra Leone, CHWs were required to support their communities to respond to the outbreak, drawing from their experiences during the Ebola outbreak [10]. With the onset of the pandemic in 2020, the Government of Sierra Leone (GoSL) released an interim guidance which identified the specific role of CHWs in the pandemic context and outlined adaptations to their role and support needed to maintain continuity of essential services and ensure an effective response to COVID-19 (Government of Sierra Leone COVID-19 Response: Interim Guidance for Community Health Workers (CHW) Program in COVID-19 context, Unpublished). This interim guidance (2020) also made provisions for CHWs to be rewarded with financial and non-financial incentives as motivation and enablers for their work. Their tasks included contact tracing, home visits to people in quarantine, community engagement and sensitization to provide key COVID-19 prevention messages to communities, and referral of people with suspected COVID-19 to isolation and treatment centres, in addition to their routine responsibilities (Government of Sierra Leone COVID-19 Response: Interim Guidance for Community Health Workers (CHW) Program in COVID-19 context, Unpublished).

There is little evidence on the experiences of CHWs in their response to COVID-19 at the community level in Sierra Leone [10]. Learning from how CHWs adapted their role and were assisted is critical to supporting CHWs in Sierra Leone and in other settings as they navigate new and existing challenges. This paper, therefore, explores the question: how did CHWs adapt their roles during the COVID-19 pandemic in Sierra Leone and what support did they receive from their families, community and health system? The research objectives are:

1. To document and analyse the adaptations made by CHWs in Sierra Leone to fulfil their roles during the COVID-19 pandemic.
2. To explore the types of support received by CHWs from their families, communities, and the health system in responding to COVID-19, with a focus on incentives, training, and supervision.
3. To provide insights and recommendations for improving the role and support of CHWs in pandemic responses, both in Sierra Leone and other similar settings.

## Methodology

### Study design

We conducted a qualitative exploratory study using key informant interviews (KIIs) at the district level with district and community level managers and Focus Group Discussions (FGDs) with CHWs (Supplementary file_1).

### Study sites

We selected two districts following a stakeholder engagement exercise and discussions between the research team and the CHW Hub in the Ministry of Health and Sanitation. Bonthe District is in the Southern Province, is hard to reach, riverine with several islands, and was less affected by the Ebola outbreak. Kenema District in the Eastern Province, is large with urban and rural areas and was heavily affected by the Ebola outbreak.

## Methods

### Key informant interviews

We purposively sampled key informants who were knowledgeable about the work of CHWs and had direct experience of managing and supervising CHWs in their communities. We intended to have a balanced representation of both female and male participants. We interviewed a total of 8 key informants (see Table 1 for more details). They included one district health management team (DHMT) representative, one CHW Peer Supervisor, One Section Chief and one Mammy Queen (Chairlady) from each district. We conducted the interviews in December 2020. The interviews were face-to-face, following strict COVID-19 safety protocols. We developed and piloted a topic guide for the interviews with questions around CHW roles and responsibilities during COVID-19, the management and support that they received, and recommendations. The lead author led the interviews in the local Krio language, with a research assistant taking notes. The interviews lasted between 30 and 90 min and were recorded following consent of participants.

### Focus group discussions

We conducted two FGDs in each district in December 2020: one with women CHWs and one with men CHWs (see Table 1 for more details). We selected CHWs based on their work experience including practical knowledge and experience of working during the COVID-19 pandemic and willingness to participate in the study. The district CHW focal person contacted CHWs to participate in the study. We developed a topic guide that included questions on their responsibilities during COVID-19, their experiences and motivations for working, the support received and challenges faced, and recommendations for further support. The topic guide was tested with several CHWs in health facilities in Freetown and adapted before being used in the study districts. The lead author facilitated the discussions in Krio language and notes were taken by the research assistant. FGDs were conducted at a guest house in Mattru Jong, Bonthe District and the DHMT Office in Kenema District and lasted between 120 and 150 min. They were recorded following the consent of participants.

**Table 1:** Participants for interviews and FGDs

	Focused Group Discussion with CHWs			Key Informants Interview			All Participants
Study Site	Men	Women	Total	Men	Women	Total	**Total**
Bonthe	7	8	15	3	1	4	**19**
Kenema	8	8	16	2	2	4	**20**
Total	**15**	**16**	**31**	**5**	**3**	**8**	**39**

### Data analysis

All recorded interviews were translated and transcribed into English for analysis. We used the thematic framework analysis approach [15] supported by NVIVO software. We developed a coding framework from the research objectives, themes emerging from reading the transcripts, whilst being guided by the interim guidance 2020 for CHW programme in COVID-19 context (Government of Sierra Leone COVID-19 Response: Interim Guidance for Community Health Workers (CHW) Program in COVID-19 context, Unpublished). We applied the coding framework to the transcripts, extracted the data for each code, and developed charts for each code. We identified and agreed key themes through virtual meetings and e-mail exchange to ensure rigour and reliability of the analysis. .

### Ethical issues

We obtained ethical approval from the Scientific and Ethics Review Committee, Ministry of Health and Sanitation, Sierra Leone (dated 9 October 2020) and from the Research Ethics Committee at the Liverpool School of Tropical Medicine (20–070) (dated 10 November 2020). Before each interview and FGD we fully explained the study using participant informant sheets, discussed any questions, and received written consent. Rigorous mechanisms to assure confidentiality in data collection, analysis and storage were followed. We followed strict COVID-19 safety measures during face-to-face interviews and FGDs, including hand washing, physical distancing and wearing of face masks.

## Results

Using the interim policy for the CHW programme as a framework (Government of Sierra Leone COVID-19 Response: Interim Guidance for Community Health Workers (CHW) Program in COVID-19 context, Unpublished), we present the findings under two main questions: 1) how did the role and responsibilities of CHWs adapt during the COVID-19 pandemic and what actions were taken to support as proposed by the GoSL interim guidance (2020)^15^? − 2) How were CHWs supported by families and communities in their adapted role?

### How did the role and responsibilities of CHWs adapt during the COVID-19 pandemic and what actions were taken to support as proposed by the GoSL interim guidance (2020)?

In this section we first present how CHWs’ role adapted during the pandemic, and second, how they were supported by the health system to undertake these new and existing responsibilities.

## Role and responsibilities of CHWs in COVID-19 response

### New role and responsibilities

During the pandemic, in both districts, CHWs took on additional responsibilities related to COVID − 19. The Government purposely involved CHWs in the response because of the inadequate numbers of formal health workers, their in-depth knowledge of their communities and their ability to effectively communicate with community members. They also have knowledge of disease prevention at the community level. As there was a high work burden for the surveillance team, tasks of contact tracing and surveillance were shifted to CHWs.

*“Technical staff are not enough… CHWs have been trained in the community and they know their people… the community respects them and believes whatever they say to them so sensitizing the community will be much easier.” (Bonthe, Key Informant, District, Male)*.

*“Because the workload was too much on the surveillance team, serving as both contact tracers and surveillance officers, so the government thought it fit to call the CHWs, go through training to know what they will have them do.” (Kenema, Key Informant, CHW Peer Supervisor, Female)*.

CHWs reported that they were asked to be part of the response by the district COVID-19 response team (DICOVEREC). CHWs were recognised as being able to easily identify people from outside their communities which can help prevent virus transmission and are strategically placed to support sensitization about the virus.

*“Why they involved us in this COVID-19 is because we have been working in our community as CHW… we know them all, tell them what to do and they will take it as gospel truth… because already we have been interacting with them. So DICOVERC thought it fits to involve us.” (Bonthe, CHW, Female)*.

CHWs used their experience working in previous outbreaks to have the confidence and motivation to support the response to COVID-19.

*“…so we used the lessons from Ebola to attack the COVID_19 outbreak … We were trained on and provided with information on how to hand wash, use of face masks… thermometer… it was not difficult on some of us as were already have the experience from the Ebola response on how to use these equipment…”. (Kenema, CHW, Male)*

*“We didn’t want the sickness to spread the way Ebola spread in our community… we have already had the know-how… so we sensitized people and tell them what to do for them not to get the disease… so that is the reason”. (Bonthe, CHW, Female)*

In the periodic lockdown phase, CHWs conducted house-to-house visits and together with Peer Supervisors (i.e., CHW with additional supervisory responsibilities of other CHWs) served as surveillance officers helping with active COVID-19 case identification, isolation and contact tracing. In addition, they had responsibilities within quarantine homes which included: monitoring temperatures of isolated individuals, looking for symptoms of the virus, and recording information on reporting forms. CHWs would often visit 10–15 quarantine homes a day. Their workload depended on the number of people with COVID-19 and number of quarantine homes in their community.

*“The workload was much… we have to visit quarantine homes twice a day and talk to them and check their temperature… And in some households, there are many people.” (Bonthe, CHW, Female)*.

They also played a significant role in community engagement and risk communication. They acted as social mobilizers sensitising people in the community, schools, motor parks, and marketplaces about the use of facemask, hand washing and social distancing.

*“Our role in the first three months of the COVID-19 outbreak was on community sensitization… on how to hand wash and use of face mask… we were doing that until the initiation of the lockdown….” (Kenema, CHW, Male)*.

They also provided informal psychological support by counselling people with COVID-19 and encouraging people to engage in best practices to avoid infection and to comply with the disease control measures set by the Government.

All participants reported that there were no gender differences in their roles and responsibilities.

*“There is no difference in the role and responsibilities (between male and female CHWs). We do the same job equally.” (Kenema, CHW, Male)*.

### Continuing routine health services during COVID-19

In addition to their specific aforementioned COVID-19’s roles and responsibilities, CHWs maintained their routine work during the COVID-19 response, which increased the already heavy workload. In both districts, this largely centred around maternal and child health including for example, monitoring and counselling of pregnant women and lactating mothers, community-based surveillance, nutritional surveillance, integrated community case management, monitoring new-born babies for immunization, and sensitization of community members on hygiene practices. They also facilitated referral of pregnant women and lactating mothers for healthcare in order to minimize complications due to pregnancy and home delivery, and maternal and newborn mortality.

*“…workload was excess…working as contact tracers, surveillance adding that to our normal CHW routine was too much…” (Kenema, CHW, Male)*.

## Ensure safe interaction for CHWs

### Avoid community level service delivery approaches that entail large gatherings of people – the “no touch policy”

The “no touch policy” was implemented during the pandemic to prevent the spread of the virus, but some CHWs found it difficult to maintain physical distance due to limited space in quarantine homes and when visiting homes to trace contacts.

*“Physical distance was hard to maintain… so I had to be really careful… I had to be a role model to other people, especially at quarantine homes.” (Bonthe, CHW, Male)*.

### Maximise the use of telehealth consultations and e-health

Promises of provision of mobile phones and monthly telephone allowance for reporting any issues that they face were made to all CHWs but were not delivered. CHWS paid out of their own pocket when using mobile phones for reporting.

*“From our own section (community), we were only promised working tools like phones and rain gears… but none were given to us…” (Bonthe, CHW, Male)*.

*“When we went for the training, we were told that transportation would take us to and from our place of work…and they will provide mobile phones for us. But we were doing the work even transportation we were not able to get. We were not able to get food, so it was out of our pocket we were providing transportation…it was out of our pocket we were having communication”. (Kenema, CHW, Female)*

*“Although they told us that during the training, they were going to give us a telephone, they didn’t do it… we continued the work. We didn’t stop… There were so many challenges but yet we work as contact tracers”. (Bonthe, CHW, Female)*

### Use of personal protective equipment (PPE)

During the COVID-19 pandemic, all health workers, including CHWs, were at increased risk of contracting the virus. To ensure their safety, the guidelines proposed cloth masks and hand sanitizers for all CHWs, and N95 masks for contact tracers and CHWs visiting people in isolation and quarantine homes. However, in reality, CHWs were not well protected.

All CHWs reported severe shortages of face masks and gloves. These shortages had several implications. First, CHWs were either delayed or not able to provide their services. Second, community trust and cooperation was reduced, as some community members accused CHWs of keeping the materials for themselves and their families. Third, CHWs felt unable to protect themselves from contracting COVID-19 and passing it on to their family and this caused huge amounts of stress.

*“We were not protected… we were risking our lives to talk to the people… they didn’t give us anything to protect ourselves… so that is what I didn’t enjoy.” (Bonthe, CHW, Female)*.

*“… during Ebola, they were giving us gloves but this time around they did not give us anything…” (Bonthe, CHW, Female)*.

*“Due to shortage of materials, some community members were assuming that we as CTC providers were the one embezzling supplies…So that created some amount of mistrust.” (Bonthe, CHW, Male)*.

### Enabling environment

All CHWs reported shortages of other essential equipment such as thermometers, soap and buckets, case investigation forms, and megaphones for sensitisation activities within the community. Regarding support from partners and other organizations to the health system,, there were some reports of local NGOs provided some assistance after recognizing the severity of the outbreak in Bonthe district. They supplied materials such as thermometers and handwashing supplies that were then distributed to accessible locations.

*“After realizing the intensity of the outbreak, we started getting support from some other local NGOs…materials like thermometer and hand washing materials were provided to us… we distributed those at strategic points that could be easily accessed by people to use.” (Bonthe, CHW, Male)*.

However, in Kenema, little support to the CHW program at the district level from development partners and NGOs was captured in this study.

It was also captured in Bonthe that some CHWs bought some of the needed supplies to enable them to continue their role during the COVID-19 response and serve their communities.

*“We didn’t receive any equipment… we buy the facemask out of our pocket … I was just working for the love I have for my people but nothing else…” (Bonthe, CHW, Female)*.

Thus, CHWs reverted to improvisation such as making handwashing stations from jerry cans and soap from firewood ash, paying for equipment from their own pockets or sharing equipment.

*“Five of us were using a single thermometer… we were using it by turn… one uses it for a few hours… then give to the other and so on… it caused a lot of delay in our work… especially in quarantine homes.” (Kenema, CHW, Male)*.

*“It affected us because we were not having our equipment, everything we have to take from our pocket… and that slows down our work.” (Bonthe, CHW, Female)*.

## Continue monitoring, reporting and supervision

Generally, CHWs report on a monthly basis but this changed during the pandemic to daily reporting of Community-Based Surveillance (CBS), sensitization, and daily field activities, like contact tracing and case investigation, which also added to their heavy workload. However, the quality of reports was not good in the two districts as a result of low literacy level or workload.

*“…some are not literate so it’s difficult for them to write report and report involves more of writing”. (Bonthe, CHW Peer Supervisor, Male)*

*“…some of the providers [CHWs] do not report accurate information due to the large size of the community they have to cover…” (Kenema, CHW focal person, Female)*.

In addition, despite the interim guidance document calling for the use of electronic reporting systems, CHWs continued to use paper-based reporting tools, reviewed by CHW peer supervisors, who then collate and report to health facility managers.

*“We have been supervised by our partners, especially those helping us in our response… we are given forms to fill every day in order to determine whether we are doing our job or not.” (Bonthe, CHW, Male)*.

These forms were not always available as it was captured that in some cases CHWs had to self- fund reproducing supplementary copies of the reporting tools.

*“…even the reporting tool was just a single copy…I had to make extra copies out of my own pocket…” (Kenema, CHW, Male)*.

CHWs were supervised by disease surveillance team members, peer supervisors and focal persons on how to correctly fill-in data collection forms. These forms were then submitted to the DICOVERC and finally fed into the response at the national level. All supervision was done face-to-face as travel restrictions did not apply to frontline workers including CHWs.

*“We were supervised on what we were trained… like CBS, sensitization… to know whether we are really implementing what we were trained in our various communities…and also based on various activities we undertake… sometimes they will even go to communities unannounced to find out whether we are really doing our job… and that is confirmed by community members that indeed they have been told what to do to keep themselves protected” (Bonthe, CHW, Male)*.

CHWs were positive about the supervision that they received as it increased their knowledge, helped them do their work better and enhanced trust among community members. As one CHW explains:

*“It (supervision) gives us weight in doing our job… people seeing us supervised serves as a boost to our job… they can easily accept whatever we tell them…It also keeps us on our toes in doing our job the right way.” (Bonthe, CHW, Male)*.

One gendered issue related to supervision was that male CHWs sometimes did not comply with directives or guidance from female peer supervisors.

*“Since I am a woman, some will make like they know it all, because I am a woman, I should not be a peer supervisor for them.” (Kenema, CHW Peer Supervisor, Female)*.

## Provide training for CHWs

All CHWs reported receiving face-to-face training on COVID-19 which included: social mobilisation, community sensitisation, contact tracing, case investigation, counselling people with COVID-19, managing homes in quarantine, and how to address health emergencies. Training was informed by the GoSL guidelines 2020.

*“We are trained first on how to address health emergency issues…we were also trained on how to counsel those infected… So, I believe if you implement what you have been trained…you will definitely enjoy the work you do, provided you know how to do your work” (Bonthe, CHW, Male).*

Training was provided by NGOs in the districts such as Partners in Health in Bonthe District, and International Rescue Committee in Kenema District. All CHWs perceived that the training was useful in helping them understand more about the illness and how to carry out their roles in the community. However, some CHWs said the training was too short. CHWs did not report any e-learning platforms.

*“It helped us understand a lot about the disease… we now understand what social distance is, use of face mask and temperature check… patient referral is also another… a lot of awareness has been created among community members.” (Bonthe, CHW, Male)*.

CHWs received visual aids like manuals, posters, and booklets to educate the community on COVID-19 prevention, including handwashing and proper face mask use. “*Even if you want to forget what you have been taught, when you watch the posters, you will remember.” (Kenema, CHW, Female)*.

## Provide remuneration and other incentives for CHWs

Government guidelines proposed a standardized remuneration package stating that all CHWs involved in the COVID-19 response should be given Le 200,000 (equivalent of $20 at the time) per week. Payments were often late, and most CHWs perceived the amount as too small compared with their workload, the level of responsibility and risk.

*“We had later payments, but we did not receive our two months backlogged, as I was one of the forty people who did not receive my stipend for the first two months.” (Kenema, CHW, Male)*.

*“The money that they gave us was too small; during a lockdown, we have to buy things in the house to eat. The family burden is too much on us we have our children to look after and other family members.” (Bonthe, CHW, Female)*.

Lockdown, travel restrictions and increased workload hindered additional income-generating activities which CHWs usually undertake to support their families. In addition, there was reported acute shortage of food, which resulted in the increase in the price of food and other staple goods, which translated into increased the stress for some CHWs. It was particularly challenging for women CHWs who were widows or single parents. All CHWs reported that they were not given other incentives, like food and non-food items, for their work in the COVID-19 response.

There were also no transport allowances for CHWs which impeded their movement around their communities, often resorting to either walking long distances or paying out of pocket to use motorcycles. It was also captured that due to unfulfilled promised incentives, some CHWs were uncooperative in fulfilling their assigned roles and responsibilities.

*“Because they do not have their incentives as promised, they do not cooperate with their given roles and responsibilities.” (Bonthe, Key Informant, CHW Peer Supervisor, Male)*.

It was also captured that female CHWs were further challenged in using the latter due to safety concerns.

## Provide mental health support

The interim guidance inferred that CHWs might experience stress, stigmatization and isolation from the communities they serve during outbreaks, and therefore, psychosocial support should be provided. However, specific mental health interventions were not described. All CHWs reported experiencing some degree of mental stress during the COVID-19 pandemic, for example social isolation and livelihood disruption. Some were traumatized by their female dependents becoming pregnant as a result of school closures, which added to their burden and affected their ability to perform their job.

*“I was really traumatized due to the pregnancy of my younger sister…life was so stressful for me due to additional burden…I have to take care of both the mother and the child…My job was affected due to stress…no help from anywhere else…I have now got more burden on me with less income.” (Kenema, CHW, Male)*.

CHWS also experienced stigma from both the community and family members due to their involvement in the COVID-19 response.

*“We were bashed at in some communities…sometimes we cannot even do our work… as most were claiming that we were disease carriers…coupled with a lot of provocation so that led to serious mental health issue amongst us.” (Bonthe, CHW, Male)*.

*“Stigma was also real…we could not even socialize with others…” (Kenema, CHW, Male)*.

In addition, as aforementioned, the lockdown halted other income sources for CHWs, and food shortages heightened mental stress.

Despite complaints to their managers, CHWs did not receive any mental health support, even though there is one mental health nurse in each district. They were advised by their managers to ignore criticisms from community members and continue doing their work.

## How were CHWs supported by families and communities in their adapted role?

In this section, we describe the support provided to CHWs from families and the community.

### Support from families

Most CHWS reported that their families were supportive of their work in the COVID-19 response. Family members gave them words of encouragement to continue the work that they were doing, whilst advising them to remain alert and careful at all times, especially when visiting quarantine homes.

*“My family and friends supported me, and they encourage us to do the work… They listen to whatever we tell them to do that alone is a big support, because they are making our work easy.” (Bonthe, CHW, Female)*.

However, there were a few examples of where CHWs received little family support. For example, one female CHW reported being distanced for fear of contracting the virus.

*“…I am not having support from anybody… my brothers are alive… one of my brothers who knows that I am a contact tracer has distanced me.” (Kenema, CHW, Female)*.

### Support from community leaders and groups

Community leaders and other community stakeholders instituted laws that supported CHWs to work outside of their home communities during the response, which helped their entry into and acceptance by the community.

*“My gratitude goes to our community leaders… they were really giving us the zeal and motivation to do our job… It made my job easy… there were no confrontations from any quarantine homes in my community.” (Kenema, CHW, Male)*.

Some CHWs reported that community leaders and existing support groups for men and women, village development committees, were supportive of CHWs’ work during the response and often applauded their efforts. They encouraged community members to listen to CHWs and follow their advice.

*“In my community, they were not supporting me financially but what I was telling them to do, they will do it… They do accept anything I say… They will do anything I asked of them and that gives me the zeal to work in the community.” (Kenema, CHW, Female)*.

*“We have got a lot of praise from community leaders…our work did not go unnoticed… we were really recognized by leaders that our work is saving our communities… our wok was also recognized by some community members, women especially, that we have been saving lives and people from different diseases in our communities.” (Bonthe, CHW, Male)*.

However, some CHWs reported being ostracised by their communities due to their involvement in the response. Some reported being isolated from all social activities in the communities as they were seen as carriers of the virus. Some CHWs felt unappreciated as their response activities were not accepted or taken up by communities due to misconceptions about the virus and accusations of prolonging and monetising the response. CHWs who had to work outside of their home communities faced the additional challenge of rejection by these communities.

*“Some of us are tenants…so, flat owners are assuming that we are also playing a huge part in prolonging the period of the disease in communities…In communities, we are only regarded by old people… whiles young people are accusing us of monetizing the whole response program…that creates a kind of stigma around us in our communities.” (Bonthe, CHW, Male)*.

Certain experiences had gendered dimensions as well. It was noted that some community members exhibited reluctance to heed female CHWs unless accompanied by a male CHW.

*“Communities do not tend to often listen to women in certain situations due to cultural beliefs…they are not given the audience they need…so in some cases we provide them with a male back up if there should be pressing issues to be addressed.” (Kenema, Key Informant, District, Male)*.

## Discussion

This study examined the adaptations of the role and responsibilities of CHWs in response to the COVID-19 pandemic in Sierra Leone. Our findings show that CHWs at the request of the GoSL, quickly took on COVID-19 role which included surveillance, contact tracing, social mobilization, and provision of psychosocial support. CHWs were trusted with these responsibilities as they were recognized as being knowledgeable about the community, were able to communicate effectively with community members and had experience of dealing with other outbreaks. In Sierra Leone and in other settings, CHWs have been useful in dealing with disease outbreaks and making health systems stronger, by building bridges between community and formal healthcare systems [8, 10, 16].

Our study found that CHWs continued to provide routine services, mostly focusing on maternal and child health, supporting a continuum of care. Maintaining essential services during a crisis is critical, as learning from other outbreaks has demonstrated [4–7]. For example, during the Ebola epidemic, the number of women delivering in health facilities fell by 80%, child immunizations drastically reduced, and access to services resulted in increased number of deaths for malaria, HIV/AIDS and tuberculosis [16].

The GoSL developed an interim guidance for the CHW programme in 2020 during the COVID-19 pandemic (Government of Sierra Leone COVID-19 Response: Interim Guidance for Community Health Workers (CHW) Program in COVID-19 context, Unpublished). Adaptations to the role of CHWs in the two districts were similar, as informed by the interim guidance. They had the same training supported by different partners in each district e.g., Partners in Health in Bonthe District, and International Rescue Committee in Kenema District. They faced the same challenges of workload during the COVID-19 response, lack of remuneration and incentives, lack of PPE, poor transportation, and stigma and other mental health issues.

Our study has shown that CHWs received some support to take on these new responsibilities as outlined in the interim guidance. These included training, provision of posters and booklets that include useful information about the pandemic, in addition, some received supplies and materials from local NGOs and international organisations such as thermometers and handwashing supplies. However, there were areas where support needed to be strengthened. Other studies also showed that with the start of the COVID-19 pandemic, most of the CHWs in Sierra Leone received training supported by UNICEF [4, 17].

During outbreaks workload of health workers increases dramatically requiring additional work hours and compensation. 5 During both the Ebola outbreak and COVID-19 pandemic, CHWs suffered heavy workloads in comparison to remunerations and risks of infection [4, 8, 10, 18], which is in keeping with our study findings. Local health governments are, therefore, encouraged to engage communities earlier in the outbreak response, recruit and train more local staff and involve local people to build response structures that could better support the existing CHWs and alleviate the workload. 6, 8, 18.

The safe interaction guidance should have meant more protection for CHWs. Similarly, during the Ebola outbreak, WHO and UNICEF, in collaboration with GoSL, developed a “no touch policy” that encouraged CHWs to avoid physical contact with patients and depend on patient history and observations [18]. In reality, and similar to the situation during Ebola outbreak, there was a shortage of PPE for CHWs in Sierra Leone, which coupled with long working hours, increased the risk of contracting the virus [4]. Other studies have indicated that most countries, especially in sub-Saharan Africa, did not prioritize CHWs in their PPE allocation in order to ensure sufficient PPE for other key providers. CHWs were given the option to stay at home or do their job unprotected putting them and their families at greater risk of infection [19–22].

According to the interim guidance, CHWs and their supervisors require sufficient training and supportive supervision for community sensitisation, awareness, and risk communication (Government of Sierra Leone COVID-19 Response: Interim Guidance for Community Health Workers (CHW) Program in COVID-19 context, Unpublished). Usually, CHWs receive periodic training and supervision related to their routine work. CHWs in Sierra Leone were satisfied with the quality of the training they received early in the pandemic and thought it was useful for maintaining effective service delivery. However, they highlighted the need for more frequent and extended training. To address this, the CHW hub at the Ministry of Health and Sanitation is currently undertaking initiatives to promote regular refresher training for all CHWs, prioritizing advocacy and resource mobilization (author discussions with CHW Hub). The aim, as it was stated in the revised national CHW policy 2016-20, is to conduct refresher training sessions for CHWs at least once every two years [11]. Additionally, efforts are underway to encourage the peripheral healthcare units and DHMTs to provide monthly mentorship and coaching to CHWs. 11 Creation of digital training materials and distribution through mobile phones as well as provision of remote supervision through online platforms can be particularly useful during emergencies [23].

Supervision played a vital role in guiding CHWs’ work, increasing their knowledge, and building trust within the community. However, some issues related to gender dynamics and compliance with directives from female peer supervisors were reported as captured elsewhere [12]. Remuneration and incentives for CHWs were often delayed and deemed inadequate given their workload and level of responsibility. At the national level, the implementation of the Community Health Information System is intended to address these issues by enabling CHWs to receive performance-based payments through monthly digital reporting, shifting away from traditional paper-based verification processes conducted at various levels. Generally, supply chain, logistics, and supporting supervision for CHWs have all been disrupted during the pandemic response and therefore, CHWs felt unsupported by the health systems [5]. Likewise, supervision was a challenge during Ebola outbreak because of shortages of staff, many CHWs needed to travel long distances to attend supervision meetings and the travel costs usually exceeded their incentive [18].

CHWs in Sierra Leone depended largely on the support they received from their families and communities. Family support helped them continue to do the challenging work during the COVID-19 pandemic. Community support included bylaws to facilitate movement of CHWs, and community leaders encouraging people to follow the advice of CHWs. However, some CHWs experienced stigma, discrimination and other mental health challenges during the COVID-19 pandemic. Studies on the Ebola outbreak and other epidemics also identified stigma and discrimination as key challenges that CHWS faced, where they were socially ostracised as they were viewed as ‘carriers of the disease’ [24, 25, 4, 18]. Community participation and sensitization can help address information gaps about disease outbreaks and overcome stigma and discrimination towards CHWs [26] and can also enhance their performance and work effectiveness [27].

### Implications for policy and practice

The study findings underscore the need for enhanced support mechanisms for CHWs during health emergencies like pandemics, including timely and sufficient remuneration, access to protective equipment, training, mental health support, and incentives. Strengthening supervision and communication channels can further enhance their effectiveness during crises.

Community leaders and support groups play a vital role in advocating for and supporting CHWs, helping dispel misconceptions and reduce stigmatization. Additionally, gender-sensitive approaches should be considered to address the specific challenges faced by female CHWs.

### Limitations of the study

This study has provided valuable insights into the experiences of Community Health Workers (CHWs) during the COVID-19 pandemic in Sierra Leone. However, it is essential to acknowledge its limitations. We conducted the study at a specific point in the pandemic’s evolution, and while it offers a snapshot of CHWs’ experiences, it may not capture potential changes over time. Moreover, this study primarily represents the perspectives of CHWs and health system stakeholders, and we recognize the need to include the voices of the community and patients for a more comprehensive understanding. Triangulating data from diverse perspectives would enhance the robustness of future research. Despite these limitations, our study has offered an in-depth understanding of the experiences of CHWs in fragile settings during the COVID-19 pandemic. It has provided valuable implications for policy and practice. Moving forward, the next steps involve implementing and testing the recommended actions to further support and strengthen CHWs in pandemic response and primary healthcare delivery.

## Conclusions

The COVID-19 pandemic in Sierra Leone showcased the remarkable dedication and adaptability of CHWs, highlighting their indispensable role amid challenging circumstances. Their significance in bridging communities and healthcare systems during health crises cannot be overstated, as they served as trusted community members with localized knowledge and skills, facilitating community sensitization, contact tracing, and crucial support. Recognizing and adequately preparing these frontline healthcare providers for future health emergencies is imperative.

Policy implementation gaps have negative consequences on the otherwise commendable efforts of CHWs. Promises of essential tools and fair compensation often went unfulfilled, while chronic shortages of protective equipment and supplies compromised their effectiveness and safety. The absence of mental health support, despite their stress and stigmatization, underscores a critical policy deficiency requiring immediate action. Adequate training and robust support mechanisms are critical for CHWs, enhancing essential knowledge but also fostering confidence and competence to deliver services effectively. Ensuring regular remuneration, access to protective gear, and mental health provisions is vital for sustaining CHWs’ morale and well-being.

The narratives of CHWs’ experiences underscore the resilience and substantial influence of dedicated individuals within the healthcare continuum. This study emphasizes the importance of well-supported CHWs in pandemic response, necessitating policy improvements, comprehensive training, and robust support.

### Supplementary Information

**Additional file 1.**

## Data Availability

The datasets generated and/or analysed during the current study are not publicly available due confidentiality and ethical restrictions but are available from the corresponding author on reasonable request.

## Abbreviations

CHWs: Community health workers
DHMT: District health management team
FGDs: Focus Group Discussions
GoSL: Government of Sierra Leone
KIIs: Key informant interviews
NGOs: Non-governmental organization
PPE: Personal protective equipment
